# Clinical features and factors related to suicidal ideation in adult patients with benzodiazepine use disorder

**DOI:** 10.1192/j.eurpsy.2024.310

**Published:** 2024-08-27

**Authors:** P. Querol Clares, G. Ortega Fernandez, R.-F. Palma Alvarez, C. Daigre, M. Alijotas Capdevila, J.-A. Ramos Quiroga, L. Grau Lopez

**Affiliations:** ^1^psychiatry; ^2^Hospital Vall d’Hebron, Barcelona, Spain

## Abstract

**Introduction:**

Benzodiazepine use disorder (BUD) has been associated with the presence of suicidal ideation (SI) in general population. It seems there is an overall increase in the risk of attempting suicide due to the increase of impulsivity, rebound and withdrawal of those who use benzodiazepines(1). However, this association has been scarcely studied.

**Objectives:**

To explore the prevalence, clinical features and factors related to lifetime SI in adults with BUD.

**Methods:**

A cross-sectional study was conducted in an outpatient center for addiction treatment between 01/01/2010 and 12/31/2021. Adult patients who met criteria for active BUD were included. Patients with language barriers, cognitive impairments and those who were participating in any clinical trial were excluded. All patients were evaluated with an ad-hoc questionnaire, EuropASI (European Addiction Severity Index), BDI (Beck Depression Inventory) and HRQoL SF-36 (Health-related quality of life according to SF-36). Univariate and bivariate analyses were performed comparing BUD patients with or without SI.

**Results:**

554 patients were included (65.2% males; M age 42.6±12.6 years). SI was reported in 57.2% of the patients. Regarding the sociodemographic variables, any type of lifetime abuse was correlated with SI (67.8%, 73.5% and 77.8% of the patients with emotional, physical and sexual abuse respectively). Considering the different psychiatric features studied, having any psychiatric diagnosis increased SI up to 64%. Depressive and cluster B personality disorders were the ones with a higher presence of SI (67.1% and 68.1% respectively). Anxiety and cluster A personality disorders had also higher proportions of SI (56.1% and 58,7% respectively). Regarding the different assessment instruments used, a higher punctuation on BDI score was seen in the group of patients with SI (23.73±12.86). The scores also showed a worse perception of the mental quality of life of those people with SI, measured by HRQoL (13.76 and 36.82±31.93 in patients with SI and no SI respectively). Considering the EuropASI, there was an increased proportion of SI in those patients with a worse familiar situation (0.44±0.30), a higher alcohol consumption (0.26±0.28) and a worse psychological condition (0.48±0.24).

**Conclusions:**

The prevalence of SI in patients with BUD is significative and is related to several clinical factors. Those factors should be taken into account in daily clinical practice, research, and any health policies on suicide. Further research should be developed.

1. Dodds, T.J. ‘Prescribed benzodiazepines and suicide risk’, The Primary Care Companion For CNS Disorders 2017; 19(2).

**Disclosure of Interest:**

None Declared

